# Noradrenaline and Adrenoreceptors Promote Prostaglandin F2α Generation in Lipopolysaccharide-Exposed Endometrial Epithelial Cells of Pigs (*Sus scrofa domesticus*)

**DOI:** 10.3390/ijms26125874

**Published:** 2025-06-19

**Authors:** Barbara Jana, Jarosław Całka, Aleksandra Mówińska

**Affiliations:** 1 Institute of Animal Reproduction and Food Research of the Polish Academy of Sciences in Olsztyn, 18 Trylińskiego St., 10-683 Olsztyn, Poland; a.mowinska@pan.olsztyn.pl; 2 Department of Clinical Physiology, Faculty of Veterinary Medicine, University of Warmia and Mazury, 13 Oczapowskiego St., 10-718 Olsztyn, Poland; calkaj@uwm.edu.pl

**Keywords:** endometrial epithelial cells, lipopolysaccharide, noradrenaline, adrenoreceptors, prostaglandin F2α generation, pig

## Abstract

Severe kinds of uterine inflammation in animals cause reproductive and economic problems. Although there are changes in prostaglandin (PG) production and noradrenergic uterine innervation during endometritis, the role of noradrenaline (NA) and adrenoreceptors (ARs) in PGF2α formation is not yet fully understood. To recognize noradrenergic control of the PGF2α generation on the cellular level during endometritis, the action of NA as well as α1-, α2- and β-ARs on protein abundances of PGF synthase (PGFS) and PG 9-ketoreductase/carbonyl reductase (CBR1) in the *Escherichia coli* lipopolysaccharide (LPS)-influenced pig endometrial epithelial cells and PGF2α release from these cells were studied. The epithelial cells were exposed to LPS and NA alone; LPS with NA; LPS with agonists of α1-, α2- and β-Ars; LPS with antagonists of β1-, β2- and β3-ARs with NA; and LPS with antagonists of β1-, β2- and β3-ARs in combinations with agonists of β1-, β2-, and β3-ARs for 24 h. PGFS and CBR1 protein abundances in cells were determined by Western blotting and PGF2α medium content by ELISA. LPS alone increased CBR1 protein abundance and PGF2α release by epithelial cells in reference to the control value. NA alone exerted a stimulatory effect on PGFS and CBR1 protein abundances and PGF2α secretion. After the exposure of cells to LPS with NA together, CBR1 protein abundance, as well as PGF2α release, was higher than in response to LPS and NA alone. PGFS protein abundance was increased by LPS with NA together compared to LPS action alone. In LPS-exposed endometrial epithelial cells, NA acting by β2- and β3-ARs leads to a rise in CBR1 protein abundance and PGF2α secretion. β2-ARs also participate in the NA excitatory effect on PGFS protein abundance. The NA effect on all the parameters tested is not mediated by α1- and α2-ARs. β2- and β3-ARs mediate the stimulatory effect of NA on PGF2α generation and secretion by the LPS-exposed pig endometrial epithelial cells. The results suggest that these cells may be a significant source of PGF2α under noradrenergic stimulation in the inflamed endometrium, and NA affects processes controlled by PGF2α during endometritis in an indirect manner.

## 1. Introduction

Uterine inflammation (endometritis, metritis) frequently develops in females, including women. Severe kinds of inflammation may reduce reproductive efficiency and production profitability in farm animals [1,2,3]. This pathology is primarily evoked by bacteria, including Gram-negative such as *Escherichia coli* (*E. coli*), *Klebsiella pneumoniae* and *Pasteurella multocida* [4]. Gram-negative bacteria possess lipopolysaccharide (LPS) on their membrane surface. LPS binds to Toll-like receptor 4-CD14 complex located on endometrial cells, initiating inflammatory signaling cascades that lead to the release of inflammatory mediators—cytokines and metabolites of arachidonic acid [5,6,7,8]. The formation, development and maintenance of an inflammatory state may result from disorders in the immune defense mechanisms of the endometrium and/or contraction of the myometrium [8,9].

In cows with clinical endometritis, increased release of prostaglandin (PG)F2α from endometrial cells [10] and the amounts of this PG in the uterine fluid during pyometra and endometritis were determined [11,12]. During clinical endometritis in cows, a rise in the mRNA expression of PG-endoperoxidase synthase-2 (PTGS-2) was noted [13]. Similarly, in the repeat-breeding cows with subclinical endometritis, increased mRNA expression of this enzyme occurs, but not PGF synthase (PGFS) [14]. Endometritis in pigs provoked by *E. coli* changed the contents of PGF2α, elevated the PTGS-2 and PG 9-ketoreductase/carbonyl reductase (CBR1) expression and reduced the expression of PGFS [15,16,17,18]. Both puerperal endometritis in cows [12] and *E. coli*-evoked in gilts [15,19] resulted in a rise in the peripheral blood level of PGFM (PGF2α metabolite: 13, 14-dihydro-15-keto-PGF2α). PGF2α produced locally during uterine inflammation has an influence on the contractile activity of the uterus [20]. This PG is also important for the appearance of inflammatory signs in acute and chronic inflammatory states [21,22]. Furthermore, studies performed on cows, heifers and gilts showed that altered production and release of PGF2α in the course of uterine inflammation are crucial for the regression mechanism of corpora lutea [15,19,23,24].

The largest population of nerve fibers found in the pig uterus comes from the sympathetic part of the peripheral nervous system [25,26]. α1 (A, B, D)- and α2 (A, B, C)-adrenoreceptor (AR) isoforms and β (1, 2, 3)-AR subtypes were identified in the uteri of many animal species, including pigs, under physiological conditions [27,28,29,30,31,32,33,34]. In the gilt uterus, these receptors are expressed in cells of luminal epithelium, glands, blood vessels, and in myometrial muscular cells [31]. Catecholamines, the transmitters of sympathetic nerve fibers, are one of the many factors changing PG generation and release by the healthy uterus. Catecholamines (noradrenaline—NA, adrenaline) increased PGF2α secretion from the human myometrium via α- and β-ARs [35]. Similarly, adrenaline elevated this PG secretion from the rat uterus through β-ARs [36]. α- and β-ARs mediate the stimulatory effect of NA on PGFS and CBR1 protein abundances and PGF2α secretion from the porcine endometrial explants [37]. The role of β-ARs in NA-induced PGF2α output by the bovine endometrial cells has also been reported [38]. Some studies also show a drop in the PGF2α secretion by the rat uterus in response to NA [39], as well as the lack of significant influence of this catecholamine on PGF2α release from the porcine oviduct [40].

In response to acute endometritis in pigs, marked alterations in uterine noradrenergic innervation [41,42] and ARs expression were revealed [31]. The role of individual α1- and α2-AR isoforms and individual β-AR subtypes in the NA-induced PGF2α formation and secretion from pig endometrial explants with inflammatory reaction is known [37]. In turn, the noradrenergic regulation of the PGF2α generation on the cellular level in the course of endometritis is not yet recognized. It is assumed that, under inflammatory conditions, NA via ARs leads to changes in this PG formation and secretion from the endometrial epithelial cells. Understanding the inflammatory link between NA, its receptors and PGF2α will lead to better indicators for preventing and treating uterine diseases. Given this, the contribution of ARs (α1 and α2 isoforms, β subtypes) in the control of NA-affected PGFS and CBR1 protein abundances and PGF2α secretion by the *E. coli* LPS-exposed pig endometrial epithelial cells were investigated.

## 2. Results

### 2.1. Impact of NA, Agonists and Antagonists of ARs on the Abundance of PGFS Protein in the LPS-Stimulated Epithelial Cells of the Endometrium

#### 2.1.1. AR Agonists and Antagonists Alone

NA increased the PGFS protein abundance compared to the control value (*p* < 0.01) and actions of α1- (*p* < 0.05), α2- (*p* < 0.01)-, β1- (*p* < 0.05) and β3 (*p* < 0.01)-AR agonists and β1-, β2- (*p* < 0.01) and β3 (*p* < 0.05)-AR antagonists (Table 1). Similar effects (*p* < 0.001) were evoked by β- and β2-AR agonists in relation to the control values and actions of the above-mentioned agonists and antagonists. After using β- (*p* < 0.05) and β2-AR (*p* < 0.001) agonists, the PGFS abundances were also higher than in response to NA.

#### 2.1.2. LPS Alone, NA Alone, LPS with NA or AR Agonists, Antagonists

**Figure 1:**
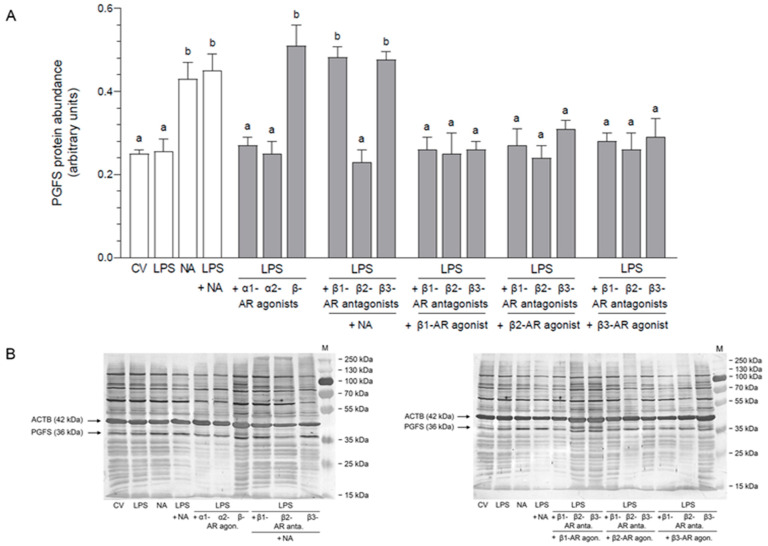
Impact of noradrenaline (NA, 10^−5^ M) alone or agonists of α1-, α2- and β-adrenoreceptors (ARs, 10^−4^ M) or antagonists of β1-, β2- and β3-ARs (10^−4^ M) with NA or antagonists of β1-, β2- and β3-ARs (10^−4^ M) in combinations with agonists of β1-, β2-, and β3-ARs (10^−4^ M) on the protein abundance of prostaglandin F synthase (PGFS) in the lipopolysaccharide (LPS, 10 ng/mL of medium)-exposed pig cultured epithelial cells of the endometrium. Enzyme abundances were determined by Western blotting, and values were normalized against β-actin (ACTB) protein abundances. Results are given as mean (±SEM) values. Bars with various letters (a, b) are statistically different (*p* < 0.05–0.001). CV: control value; agon.: agonist; anta.: antagonist (**A**). Demonstration blots for PGFS and ACTB, with bands for each treatment. For the PGFS antibody, bands are present at 36 kDa, and for the ACTB antibody at 42 kDa. M: marker (**B**).

The increased PGFS protein abundances, in relation to the control value and LPS influence, were noted after exposure to NA, LPS with NA (*p* < 0.01), LPS with β-AR agonist (*p* < 0.001) and LPS combined with β1- (*p* < 0.001) and β3-AR (*p* < 0.01) antagonists combined with NA (Figure 1). When comparing actions of NA and LPS with NA, the protein abundances of PGFS were lower (*p* < 0.01) than under the influence of LPS with α1- and α2-AR agonists, LPS with β2-AR antagonist with NA, LPS with β1-, β2- and β3-AR antagonists with β1-AR agonist, LPS with β1- (*p* < 0.001), β2- (*p* < 0.05) and β3-AR (*p* < 0.001) antagonists with β2-AR agonist, and LPS with β1-, β2- (*p* < 0.001) and β3-AR (*p* < 0.05) antagonists together with β3-AR agonist.

### 2.2. Impact of NA, Agonists and Antagonists of ARs on the Abundance of CBR1 Protein in the LPS-Stimulated Epithelial Cells of the Endometrium

#### 2.2.1. AR Agonists and Antagonists Alone

The protein abundance of CBR1 was augmented in response to NA and β-AR agonist in reference to the control value (NA: *p* < 0.01; β-AR agonist: *p* < 0.001) as well as compared to the influences of α1-, α2- and β1-AR agonists and β1-, β2- and β3-AR antagonists (*p* < 0.001) (Table 1). β2- and β3-AR agonists increased the abundances in relation to the control value (*p* < 0.001) and actions of NA (*p* < 0.01), α1-, α2- (*p* < 0.001), β- (*p* < 0.05) and β1-AR (*p* < 0.001) agonists and β1-, β2- and β3-AR antagonists (*p* < 0.001).

#### 2.2.2. LPS Alone, NA Alone, LPS with NA or AR Agonists, Antagonists

**Figure 2:**
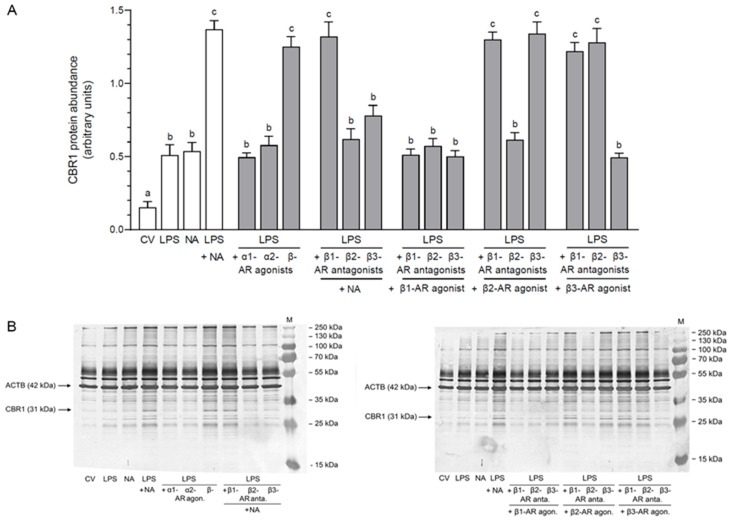
Impact of noradrenaline (NA, 10^−5^ M) alone or agonists of α1-, α2- and β-adrenoreceptors (ARs, 10^−4^ M) or antagonists of β1-, β2- and β3-ARs (10^−4^ M) with NA or antagonists of β1-, β2- and β3-ARs (10^−4^ M) in combinations with agonists of β1-, β2- and β3-ARs (10^−4^ M) on the protein abundance of prostaglandin 9-ketoreductase/carbonyl reductase (CBR1) in the lipopolysaccharide (LPS, 10 ng/mL of medium)-exposed pig cultured epithelial cells of the endometrium. Enzyme abundances were determined by Western blotting, and values were normalized against β-actin (ACTB) protein abundances. Results are given as mean (±SEM) values. Bars with various letters (a, b, c) are statistically different (*p* < 0.05–0.001). CV: control value; agon.: agonist; anta.: antagonist (**A**). Demonstration blots for CBR1 and ACTB, with bands for each treatment. For the CBR1 antibody, bands are present at 31 kDa, and for the ACTB antibody at 42 kDa. M: marker (**B**).

Compared to the control value, an increase in the CBR1 protein abundances occurred in response to LPS (*p* < 0.05); NA (*p* < 0.01); LPS with NA (*p* < 0.001); LPS with α1- (*p* < 0.05), α2 (*p* < 0.01), and β-AR (*p* < 0.001) agonists; LPS with β1-, β2- and β3-AR antagonists with NA (*p* < 0.001); LPS with β1- (*p* < 0.05), β2- (*p* < 0.01) and β3-AR (*p* < 0.05) antagonists with β1-AR agonist; LPS together with β1-, β2- and β3-AR antagonists with β2-AR agonist (*p* < 0.001); and LPS together with β1-, β2- (*p* < 0.001) and β3-AR (*p* < 0.05) antagonists combined with β3-AR agonist (Figure 2). The enzyme abundance after using LPS combined with NA was increased (*p* < 0.001) compared to individual LPS as well as NA actions. α1- and α2-AR agonists in the LPS-treated cells decreased (*p* < 0.01) the CBR1 abundances in relation to influence of LPS with NA. The abundance of enzyme was elevated (*p* < 0.001) by β-AR agonist in reference to separate LPS and NA effects. In the LPS-treated cells, β1-AR antagonist together with NA led to an increase (*p* < 0.001) in the CBR1 abundance compared to LPS and NA influences. β2- and β3-AR antagonists combined with NA reduced (*p* < 0.001) the abundances of CBR1 in these cells compared to the influence of LPS with NA. The enzyme abundances in the LPS-exposed cells were dropped (*p* < 0.001) by β1-, β2- and β3-AR antagonists combined with β1-AR agonist in relation to LPS with NA mutual action. LPS with β1- and β3-AR antagonists with β2-AR agonist elevated (*p* < 0.001) the CBR1 abundances compared to LPS and NA influences. β2-AR antagonist with β2-AR agonist lowered (*p* < 0.001) the enzyme abundance in the LPS-treated cells versus LPS with NA effect. The enzyme abundances in the LPS-treated cells were increased (*p* < 0.001) after adding β1- and β2-AR antagonists combined with β3-AR agonist versus individual LPS as well as NA actions. In these cells, in response to β3-AR antagonist with β3-AR agonist, the CBR1 abundance was lower (*p* < 0.001) than in response to LPS with NA.

### 2.3. Impact of NA, Agonists and Antagonists of ARs on the Concentration in the Medium of PGF2α Following Culture of the LPS-Stimulated Epithelial Cells of the Endometrium

#### 2.3.1. AR Agonists and Antagonists Alone

NA (*p* < 0.05), β-, β2- and β3-AR agonists increased (*p* < 0.01) the PGF2α medium contents compared to the control value, as well as after the application of α1-, α2- and β1-AR agonists and β1-, β2- and β3-AR antagonists (Table 1).

#### 2.3.2. LPS Alone, NA Alone, LPS with NA or AR Agonists, Antagonists

**Figure 3:**
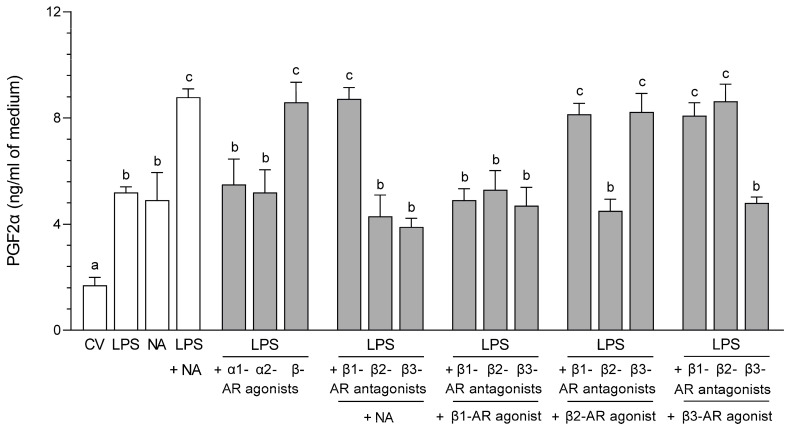
Impact of noradrenaline (NA, 10^−5^ M) alone or agonists of α1-, α2- and β-adrenoreceptors (ARs, 10^−4^ M) or antagonists of β1-, β2- and β3-ARs (10^−4^ M) with NA or antagonists of β1-, β2- and β3-ARs (10^−4^ M) in combinations with agonists of β1-, β2- and β3-ARs (10^−4^ M) on the prostaglandin F2α (PGF2α) medium content following incubation of the lipopolysaccharide (LPS, 10 ng/mL medium)-exposed pig cultured epithelial cells of the endometrium. PGF2α contents in the medium were estimated using ELISA. Results are given as mean (±SEM) values. Bars with various letters (a, b, c) are statistically different (*p* < 0.05–0.001). CV: control value; agon.: agonist; anta.: antagonist.

Compared to the control value, an increase in the PGF2α medium contents was determined after using NA; LPS (*p* < 0.05); LPS with NA (*p* < 0.001); LPS with α1- (*p* < 0.01), α2- (*p* < 0.05) and β-AR (*p* < 0.001) agonists; LPS with β1- (*p* < 0.001), β2- and β3-AR (*p* < 0.05) antagonists with NA; LPS with β1-, β2- and β3-AR antagonists with β1-AR agonist (*p* < 0.01); LPS with β1- (*p* < 0.001), β2- (*p* < 0.05) and β3-AR (*p* < 0.001) antagonists with β2-AR agonist; and LPS with β1-, β2- (*p* < 0.001) and β3-AR (*p* < 0.05) antagonists combined with β3-AR agonist (Figure 3). The addition of LPS with NA augmented the PGF2α content versus LPS (*p* < 0.05) and NA (*p* < 0.01) actions. Exposure to LPS with α1- and α2-AR agonists reduced (*p* < 0.05) the PG contents compared to LPS with NA influence. LPS with β-AR agonist increased the PGF2α amount in reference to LPS (*p* < 0.05) and NA (*p* < 0.01) actions. After using β1-AR antagonist with NA, the LPS-treated cells responded with a rise in the PG content compared to LPS (*p* < 0.05) and NA (*p* < 0.01) influences. LPS with β2- and β3-AR antagonists with NA reduced (*p* < 0.001) the PGF2α contents in relation to LPS with NA action. Following the addition LPS with β1- (*p* < 0.01), β2- (*p* < 0.05) and β3-AR (*p* < 0.01) antagonists together with β1-AR agonist, there was a reduction in PG amounts relative to LPS with NA action. In LPS-exposed cells, β1- and β3-AR antagonists with β2-AR agonist increased (*p* < 0.05) the PGF2α contents compared to LPS and NA effects. LPS with β2-AR antagonist with β2-AR agonist decreased (*p* < 0.001) the PG content versus LPS with NA action. LPS with β1- and β2-AR antagonists with β3-AR agonist caused a rise (*p* < 0.05) in the PGF2α medium contents in reference to LPS and NA influences. After exposure to LPS with β3-AR antagonist with β3-AR agonist, the PG amount was lower (*p* < 0.01) than after exposure to LPS together with NA.

## 3. Discussion

Severe acute endometritis in pigs resulted in an increase in the population of noradrenergic uterine perikarya in both sympathetic ganglia, such as the caudal mesenteric [41] and paracervical [42]. During this pathological state, changes in the density of nerve fibers expressing dopamine β-hydroxylase (enzyme participating in NA synthesis) in the vicinity of endometrial structures [43] as well as of α1D-, α2 (A, C)- and β (1, 2)-AR abundances also occur [31]. Noradrenergic regulation mechanisms of PG generation in an inflamed uterus are not well understood. In relation to PGF2α, the significance of NA in connection with ARs in the production and secretion of this PG by the sections obtained from healthy and inflamed pig endometrium was reported [37]. Moreover, the cells of luminal and glandular epithelium, stroma and blood vessels in pig endometrium possess ARs [31]. The endometrial epithelium is also an important site for PGF2α formation under physiological and inflamed conditions [44,45,46,47,48]. In this study, cellular mechanisms of noradrenergic regulation of PGF2α synthesis during endometritis were examined using the *E. coli* LPS-stimulated epithelial cells from pig endometrium.

It was found that LPS and NA alone promoted the PGF2α secretion by pig endometrial epithelial cells. This effect of LPS was accompanied by an increase in CBR1 protein abundance and by a lack of significant change in PGFS protein abundance. After exposure of bovine endometrial epithelial cells to the *E. coli* LPS, no significant increase in the secretion of PGF2α and protein expression of PGFS was found [49]. In turn, the PGF2α release was stimulated by LPS in the equine endometrial explants [50] and by *E. coli* in the bovine uterine explants [51]. In the endometrium of pigs with *E. coli*-induced inflammation in vivo, an increase in the PGF2α secretion and CBR1 protein abundance and a decrease in PGFS protein abundance were found [17,37]. However, in the endometrium of repeat-breeding cows with subclinical inflammation, PGFS mRNA expression did not significantly alter [14]. In reference to the present study, the increased PGF2α secretion by the LPS-exposed endometrial epithelial cells may result from an increase in CBR1 protein abundance.

The excitatory action of NA on PGF2α release from the endometrial epithelial cells was coincident with the elevation in CBR1 and PGFS protein abundances. This effect was exerted by NA on the above parameters in the healthy pig endometrial sections [37]. Noradrenaline stimulated the secretion of PGF2α by the bovine endometrial cells [38]. Similarly, adrenaline increased PGF2α release from the rat uterus [36]. Additionally, both NA and adrenaline also promoted PGF2α secretion by the human myometrium [35]. On the other hand, NA dropped the PGF2α release by the rat uterus [39]. It is postulated that the stimulatory influence of NA on PGF2α release by the endometrial epithelial cells, revealed here, may be a consequence of the NA-stimulated rise in the CBR1 and PGFS protein abundances. The obtained findings expand the current information about the connections between catecholamines and uterine PGs under physiological conditions, especially with regard to the cell synthesis.

It is known that the pig endometrial stripes with *E. coli*-provoked inflammatory reaction in vivo conditions in response to NA released more PGF2α, and showed higher CBR1 protein abundance and lower PGFS protein abundance [37]. The present report shows that combined treatment of the endometrial epithelial cells with LPS and NA increased the PGF2α release and CBR1 and PGFS protein abundances versus the control values. Moreover, after using LPS and NA, the increase in the PGF2α release and CBR1 abundance was more significant than the influence of LPS and NA alone. The cells responded to LPS and NA with higher PGFS abundance compared to LPS action alone. It is difficult to explain the combined effect of LPS and NA on the particular studied parameters. Further studies are needed to elucidate the intracellular mechanisms of their actions.

The current study shows completely new data on the mediation of ARs in NA action on the PGF2α generation in the LPS-exposed endometrial epithelial cells. It was found that the non-selective β-AR agonist enhanced the CBR1 and PGFS protein abundances and PGF2α secretion by the studied cells. Further, in the LPS-exposed cells, the selective β2- and β3-AR antagonists led to a drop in the NA excitatory influence on the CBR1 abundance and PGF2α secretion. Such a situation was found in relation to the PGFS abundance after using β2-AR antagonist. Thus, it was indicated that in the LPS-exposed endometrial epithelial cells, the β2- and β3-ARs contribute to the NA influence on protein abundance of CBR1 and PGF2α secretion, as well as that β2-ARs participate in the NA influence on PGFS protein abundance. These statements were also confirmed by the treatment of epithelial cells with the selective β1-, β2- and β3-AR antagonists in combinations with selective agonists of the above β-AR subtypes. In this study, the CBR1 and PGFS protein abundances and PGF2α release by the LPS-exposed endometrial epithelial cells after noradrenergic actions were not linked to the stimulation of α1- and α2-ARs. However, in addition to β-ARs, the expression of α1- and α2-ARs also takes place in the luminal and glandular epithelial cells in the porcine inflamed and healthy endometrium [31]. As in the current study, the role of β-ARs, but not α1- and α2-ARs, was revealed in the PGI2 and PGE2 generation and release by the LPS-exposed pig endometrial epithelial cells. More precisely, β1- and β2-ARs are important for the relationship between NA and PGI2 [52], while β1-, β2- and β3-ARs mediate between NA and PGE2 (Jana et al., in review). In turn, NA through β2-ARs affected the PGF2α secretion by bovine endometrial cells under physiological conditions [38]. NA increased the CBR1 and PGFS protein abundances and PGF2α release by the inflamed and healthy porcine endometrial explants by activating not only β1- and β2-ARs but also the isoforms of α1- and α2-ARs [37]. NA and adrenaline stimulated release of PGF2α from the human myometrial layer by binding with α- and β-ARs [35] as well as adrenaline from the uterus of the rat through β-ARs [36].

The obtained indications allow for a better understanding of the regulation mechanisms of PG formation in the inflamed uterus. Namely, they significantly deepen the data on the noradrenergic control of endometrial PGF2α generation and release from endometrium under inflammatory conditions. The greater secretion of PGF2α by the porcine LPS-exposed cells after using NA, accompanied by the increased CBR1 protein abundance, shows that these cells are a significant site of PGF2α production in the inflamed endometrium. This suggest that PGF2α released from the endometrial epithelium in response to catecholamines during endometritis, acting locally, may modulate the inflammatory process by influencing vascular permeability, output of pro-inflammatory cytokines and chemokines and phagocytic activity of neutrophils [22,53,54]. In turn, the extra-uterine PGF2α importance during uterine inflammation refers mainly to the early regression of corpus luteum [15,19,23,24], as well as to prolongation of the luteal phase [49,55]. Moreover, the results of the present study concerning the contribution of particular subtypes of β-ARs in the PGF2α formation and secretion by the porcine LPS-stimulated endometrial epithelial cells may be the basis for developing drugs (agonists, antagonists of particular subtypes of β-ARs) to control the inflammatory process in the uterus and the functions of ovaries.

## 4. Material and Methods

### 4.1. Collection of Uteri from Gilts

The uteri were harvested in the local slaughterhouse within 5 min after the gilts were slaughtered. The estrous cycle phase was estimated based on macroscopic observation of the ovaries according to Akins and Morrissette [56]. The uteri were harvested from gilts (n = 4) on day 8 of the estrous cycle. Isolation of cells on this day ensures that they easily became confluent during culture. The collected uteri were placed on ice and delivered to the laboratory within 25 min.

### 4.2. Isolation of Epithelial Cells from the Endometrium

**Figure 4:**
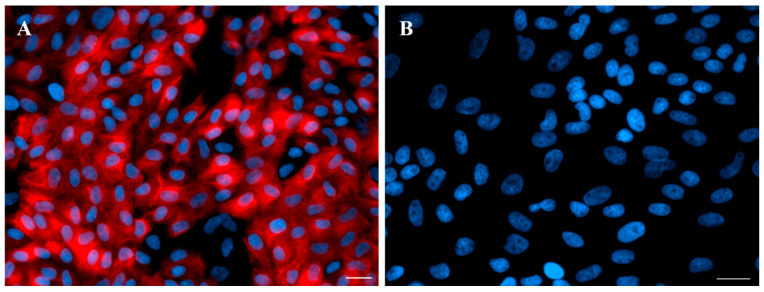
The purity of the endometrial epithelial cell culture purity checked by positive immunofluorescent staining for anti-cytokeratin (**A**) and negative anti-vimentin staining (**B**). Scale bar: 20 µm.

The epithelial cells from the endometrium were isolated according to the method given previously [57] with modifications. Uteri horns were rinsed in sterile phosphate-buffered saline (PBS; 137 mM NaCl/cat. no. 79412116, POCH, Gliwice, Poland; 27 mM KCl/cat. no. 739740114, POCH, Gliwice, Poland; 10 mM Na_2_HPO_4_/cat. no. 117992300, CHEMPUR, Piekary Śląskie, Poland; 2 mM KH_2_PO_4_/cat. no. 742020112, POCH, Gliwice, Poland; pH 7.4) supplemented with antibiotics (100 IU/mL penicillin and 100 µg/mL streptomycin; cat. no. 15140-122, Life Technologies, Bleiswijk, The Netherlands). Fragments of the horns collected from the middle parts were cut longitudinally on the surface of the mesometrium. The endometrium was separated from the myometrium by the use of scissors. The dissecting microscope was used to confirm the separation of particular layers. The small slices of the endometrium were digested using 0.2% (*w*/*v*) dispase (cat. no. 17105041, Life Technologies, Grand Island, NY, USA) in Dulbecco’s PBS (cat. no. D5773, Sigma, Kanagawa, Japan) at 37 °C for 50 min with gentle shaking. The cell suspension was filtered through a 270 µm mesh to separate the remaining endometrial parts. The released epithelial cells were resuspended with Medium 199 with 5% normal calf serum (NCS; cat. no. N4637, Sigma), pelleted by centrifugation at 200× *g* for 10 min and washed once with Medium 199 (cat. no. M5017, Sigma) containing 5% (*w*/*v*) NCS and antibiotics. Red blood cells were removed from cell suspensions using red blood cell lysing buffer (cat. no. R7757, Sigma). The epithelial cells were washed three times with fresh Medium 199 containing 5% NCS and filtered using a cell strainer with a diameter of 100 µm (Becton Dickinson, Franklin Lakes, NJ, USA). The fraction that passed through was collected. The cells were counted in a hemocytometer and seeded onto 75 cm^3^ culture flasks (2 × 10^6^ cells/mL of medium). Epithelial cells were incubated (37 °C in a humidified atmosphere—95% air and 5% CO_2_—for 5 h). Purified non-attached epithelial cells were obtained, centrifuged and suspended in fresh Medium M199 with 10% NCS, and then seeded onto the new culture flasks. The viability of cells, estimated by exclusion of 0.5% (*w*/*v*) trypan blue dye (cat. no. T6146, Sigma), was approximately 90%. The cells were immunofluorescently stained for the presence or absence of vimentin and cytokeratin, according to Zlotkowska and Andronowska [58]. Briefly, cells were seeded onto 8-chamber cell imaging coverglasses (Eppendorf, Hamburg, Germany) and cultured until reaching 70% confluence. Cells were washed with pre-warmed PBS and fixed in 4% paraformaldehyde (pH 7.45) for 20 min. Non-specific binding was blocked by 1 h incubation in blocking solution (0.1 M PBS, 0.1% BSA, 0.05% Trimerosol, 10% Normal Donkey Serum). Overnight incubation with primary antibodies for vimentin and cytokeratin (cat. no. ab20346, Abcam, Cambridge, UK, and cat. no. C1801, Sigma, respectively) was performed in 4 °C. Next day, secondary antibodies were added into each well (anti-rabbit or anti-mouse antibodies conjugated with Alexa 594, LifeTechnologies, Waltham, MA, USA), and 90 min incubation was performed in dark. Staining of cell nuclei was performed by incubation in 0.01% DAPI (Sigma Aldrich, Darmstadt, Germany) for 30 min. Plastic chambers were removed from the coverslips, and slides were mounted in VECTASHIELD^®^ Antifade Mounting Medium (Vector Laboratories, London, UK). Pictures were made with Axio Observer inverted microscope (Zeiss, Oberkochen, Germany). Observed immunofluorescent staining pattern provided evidence that epithelial cell purity was between 85% and 90% (Figure 4). During cell cultures, every 24 h the medium was changed. Before start of the experiment, the cell confluence was estimated using a Zeiss Axioplan light microscope (Zeiss, Germany) and was 80% to 90% following 5–6 days of culture.

### 4.3. Adding LPS, NA, Agonists and Antagonists of ARs to the Endometrial Epithelial Cells

Before the epithelial cells were exposed to exogenous factors, they were rinsed with fresh Medium 199 and next treated for 24 h with Medium 199 containing 2% BSA, 10% NCS and antibiotics containing no exogenous factors (control value) or with the addition of *E. coli* (O55:B5); LPS alone (10 ng/mL, cat. no. L2880, Sigma); NA alone (10^−5^ M, Levonor, Warszawskie Zakłady Farmaceutyczne Polfa, Warszawa, Poland); LPS (10 ng/mL) with NA (10^−5^ M); AR agonists alone (each at a dose of 10^−4^ M) for α1- (/R/-/-/-phenylephrine hydrochloride, cat. no. P6126), α2- (clonidine hydrochloride, cat. no. C7897), β- (isoprenaline hydrochloride, cat. no. I5627), β1- (dobutamine hydrochloride, cat. no. D0676), β2- (salbutamol, cat. no. S8260) and β3-ARs (sodium salt hydrate, cat. no. BRL37344); as well as AR antagonists alone (each at a dose of 10^−4^ M) for β1- (RS-atenolol, cat. no. 0387), β2- (ICI 118,551 hydrochloride, cat. no. 0821) and β3-ARs (SR 59230A hydrochloride, cat. no. 1511). The cells were also treated with LPS (10 ng/mL) in combination with α1-, α2- or β-AR agonists (each at a dose of 10^−4^ M); or together with β1-, β2- or β3-AR antagonists (each at a dose of 10^−4^ M) and NA (10^−5^ M); or together with β1-, β2- or β3-AR antagonists (each at a dose of 10^−4^ M) and β1-AR agonist (10^−4^ M); or together with β1-, β2- or β3-AR antagonists (each at a dose of 10^−4^ M) and β2-AR agonist (10^−4^ M); or together with β1-, β2- or β3-AR antagonists (each at a dose of 10^−4^ M) and β3-AR agonist (10^−4^ M). The agonists used were from Sigma, while the antagonists were from Tocris Bioscience. Preliminary dilutions of all exogenous factors were prepared according to the manufacturer’s indications (LPS, α1-, α2-, β-, β1-AR agonists and β1- and β2-AR antagonists were diluted in 0.2 mm-filtrated distilled water; β2-AR agonist was diluted in methanol/cat. no. 621990110, POCH, Gliwice, Poland; β3-AR agonist and β3-AR antagonist were diluted in dimethyl sulfoxide/cat. no. W387509, Sigma) and then stored at −20 °C. The same medium (as in the culture of epithelial cells) was used to prepare the final dilutions of the above factors and NA. The PGE2 secretion after treatment with a nitric oxide (NO) donor (NONOate; at a dose of 10^−4^ M, cat. no. 82150, Cayman Chemical Co., Ann Arbor, MI, USA) was applied to evaluate the epithelial cells’ reactivity. Doses of the exogenous factors, as well as the culture period, were selected based on the data of pilot experiments and in relation to the authors’ previous research. Treatments were carried out in triplicate, using cells from particular gilts (n = 4). After a period of cell culture, the medium was transferred to tubes containing 5% EDTA (cat. no. 118798103, CHEMPUR, Piekary Śląskie, Silesia, Poland), 1% acetylsalicylic acid (cat. no. 107140422, POCH Gliwice, Gliwice, Poland) solution (pH 7.4), and placed at −20 °C for PGF2α content estimation by the ELISA method. The cells were assigned to determine the abundance of PGFS and CBR1 proteins by Western blot analysis.

### 4.4. Western Blot Analysis

After the culture medium was collected, the epithelial cells were washed with PBS and lysed with 240 µL of ice-cold RIPA buffer (mmol/L Tris HCl, pH 7.4/cat. no. T150350, Sigma; 150 mmol/L NaCl/cat. no. 794121116, POCH, Gliwice, Poland; 1% Triton X-100 (*v*/*v*)/cat. no. T8787, Sigma; 0.5% sodium deoxycholate (*w*/*v*)/cat. no. D6750, Sigma; 0.1% sodium dodecyl sulphate (*w*/*v*)/SDS/cat. no. L3771, Sigma; 1 mmol/L EDTA/cat. no. 879810429, POCH, Gliwice, Poland) combined with protease inhibitor cocktail (cat. no. P8340, Sigma), and then centrifuged (5 min, 800× *g*). The supernatant was frozen at −80 °C until the study was conducted. The Bradford method [59] was used to determine the protein levels. Equal amounts of protein isolate (20 μg) were dissolved in sodium dodecyl sulphate (SDS, cat. no. L3771, Sigma), a gel-loading buffer, heated (95 °C, 4 min), and separated by 12% SDS-polyacrylamide gel electrophoresis. Separated proteins were electroblotted onto 0.45 µm Immobilon-P PVDF membranes (cat. no. IOVH00010, Sigma) in a buffer for transfer. To block non-specific binding sites, 5% fat-free dry milk (Spółdzielnia Mleczarska, Gostyń, Poland) in Tris (cat. no. T1503, Sigma)-buffered saline Tween 20 (cat. no. P1379, Sigma) buffer was used (21 °C, 1.5 h). After blocking, the incubation (4 °C, 18 h) of membranes with polyclonal rabbit antibodies for PGFS (in dilution 1:400; cat. no. AV48180, Sigma) and polyclonal goat antibody for CBR1 (in dilution 1:5000; cat. no. NB100-1066, Novus Biologicals, Centennial, CO, USA) was performed. Next, the membranes were incubated (21 °C, 1.5 h) with the following secondary antibodies: alkaline phosphatase-conjugated goat anti-rabbit for PGFS (in dilution 1:10,000; cat. no. A3687, Sigma) and alkaline phosphatase-conjugated rabbit anti-goat antibody for CBR1 (in dilution 1:10000; cat. no. NB7349, Novus Biologicals, USA). Complexes were visualized using a standard alkaline phosphatase visualization procedure (NBT-BCIP; cat. no. 72091, Sigma). All analyses were performed three times. The specificity of anti-PGSF and anti-CBR1 antibodies was present earlier in the endometrium of gilts [37]. PGFS and CBR1 membranes were reprobed using polyclonal rabbit anti-β-actin (ACTB) antibody (in dilution 1:6000; cat. no. ab119716, Abcam). Imaging and quantitation were performed using a CHEMIDOC Touch Imaging System (Image Lab 5.2, Bio-Rad Laboratories, Hercules, CA, USA).

### 4.5. ELISA Method

The PGF2α content in the medium was estimated by ELISA kits (cat. no. 516011, Cayman Chemical Co.), according to the manufacturer’s instructions. Briefly, a PGF2α standard curve, ranging from 3.9 to 500 pg/mL, was prepared. In each well of the 96-well plates, 50 µL of culture medium, PGF2α acetylcholinesterase Tracer and PGF2α antiserum were placed. Samples were estimated in duplicates. The plates were then incubated (4 °C, 18 h). After this time, Ellman’s Reagent was placed into each well, and the plates were incubated (21 °C, 2 h). The plates were read at a wavelength of 420 nm (LABSYSTEM GENESIS V 3.00, Instrument Version Multiskan ex Primary EIA V. 2.1-0). The intra-assay coefficient of variation was 5.9%, while the inter-assay coefficient of variation was 10.1%.

### 4.6. Statistical Analysis

The only data obtained from endometrial epithelial cell cultures considered were those for which PGE2 secretion in response to NONOate was statistically significant. By using the Shapiro–Wilk test, the normal distribution of data (*p* > 0.05) and the normal distribution of residuals (*p* > 0.05) were determined. The homogeneity of variances was evaluated by Bartlett’s test (*p* > 0.05). Before conducting the above tests, the PGF2α results in Table 1 were transformed logarithmically (log_10_). The results are presented as the mean (±SEM) values from four individual experiments (gilts). Statistical analyses were performed using a one-way ANOVA, followed by the Bonferroni test (InStat Graph Pad, San Diego, CA, USA). Statistical significances were defined as *p* < 0.05.

## 5. Conclusions

In the LPS-stimulated porcine endometrial epithelial cells, NA increased the PGFS and CBR1 protein abundances and PGF2α release. β2- and β3-ARs participate in the excitatory action of NA on the abundance of CBR1 protein and PGF2α secretion, while β2-ARs are activated by NA for a rise in PGFS protein abundance. During spontaneous uterine inflammation in animals and women, these ARs may be important for the upregulation of the production and release of PGF2α from the endometrial epithelium by endogenous catecholamines. The results suggest that by affecting endometrial epithelial cells, NA may indirectly influence processes regulated by PGF2α in the inflamed endometrium. The obtained findings will help in better understanding the etiopathogenesis of uterine inflammation at the cellular level, as well they may be used to develop new methods for the prevention and treatment of uterine pathologies, improving the function of the reproductive tract and the profitability of production on the farm.

## Figures and Tables

**Table 1:** Impact of noradrenaline (NA, 10^−5^ M) alone or agonists of α1-, α2- and β-adrenoreceptors (ARs, 10^−4^ M) alone or antagonists of β-ARs (10^−4^ M) alone on the protein abundances of prostaglandin (PG)F synthase (PGFS) and PG 9-ketoreductase/carbonyl reductase (CBR1) and release of PGF2α from the pig cultured epithelial cells of the endometrium. Enzyme abundances were determined by Western blotting, and values were normalized against β-actin (ACTB) protein abundances. PGF2α contents in the medium were determined using ELISA. Results are given as mean (±SEM) values. Values with various letters (a, b, c, d) in columns are statistically different (*p* < 0.05–0.001). CV: control value; agon.: agonist; anta.: antagonist.

Treatment	PGFS	CBR1	PGF2α
	(Protein Abundances, Arbitrary Units)		(ng/mL of Medium)
CV	0.25 ± 0.01 ^a^	0.15 ± 0.04 ^a^	1.72 ± 0.31 ^a^
NA	0.43 ± 0.04 ^b^	0.53 ± 0.06 ^b^	4.91 ± 1.04 ^b^
α1 agon.	0.28 ± 0.03 ^a^	0.14 ± 0.06 ^a^	1.51 ± 0.22 ^a^
α2 agon.	0.21 ± 0.04 ^a^	0.19 ± 0.03 ^a^	1.43 ± 0.25 ^a^
β agon.	0.66 ± 0.03 ^c^	0.58 ± 0.05 ^b^	6.09 ± 1.01 ^b^
β1 agon.	0.29 ± 0.03 ^a^	0.22 ± 0.03 ^a^	1.32 ± 0.51 ^a^
β2 agon.	0.83 ± 0.05 ^c^	0.81 ± 0.04 ^d^	6.31 ± 1.25 ^b^
β3 agon.	0.22 ± 0.03 ^a^	0.79 ± 0.02 ^d^	6.23 ± 1.19 ^b^
β1 anta.	0.19 ± 0.06 ^a^	0.14 ± 0.03 ^a^	1.43 ± 0.11 ^a^
β2 anta.	0.24 ± 0.03 ^a^	0.23 ± 0.02 ^a^	1.61 ± 0.22 ^a^
β3 anta.	0.26 ± 0.05 ^a^	0.17 ± 0.04 ^a^	1.52 ± 0.39 ^a^

## Data Availability

All relevant data are contained within the manuscript.
